# On the Ontology Based Representation of Cell Lines

**DOI:** 10.1371/journal.pone.0048584

**Published:** 2012-11-07

**Authors:** Matthias Ganzinger, Shan He, Kai Breuhahn, Petra Knaup

**Affiliations:** 1 Institute of Medical Biometry and Informatics, Heidelberg University, Heidelberg, Germany; 2 Department of Biomedical Informatics, University of Utah, Salt Lake City, Utah, United States of America; 3 Institute of Pathology, Heidelberg University, Heidelberg, Germany; University of Georgia, United States of America

## Abstract

Cell lines are frequently used as highly standardized and reproducible *in vitro* models for biomedical analyses and assays. Cell lines are distributed by cell banks that operate databases describing their products. However, the description of the cell lines' properties are not standardized across different cell banks. Existing cell line-related ontologies mostly focus on the description of the cell lines' names, but do not cover aspects like the origin or optimal growth conditions. The objective of this work is to develop an ontology that allows for a more comprehensive description of cell lines and their metadata, which should cover the data elements provided by cell banks. This will provide the basis for the standardized annotation of cell lines and corresponding assays in biomedical research. In addition, the ontology will be the foundation for automated evaluation of such assays and their respective protocols in the future. To accomplish this, a broad range of cell bank databases as well as existing ontologies were analyzed in a comprehensive manner. We identified existing ontologies capable of covering different aspects of the cell line domain. However, not all data fields derived from the cell banks' databases could be mapped to existing ontologies. As a result, we created a new ontology called *cell culture ontology (CCONT)* integrating existing ontologies where possible. CCONT provides classes from the areas of cell line identification, origin, cell line properties, propagation and tests performed.

## Introduction

Cell lines are well established *in vitro* tools for biomedical research including cancer and pharmaceutical research. The cell lines are usually established from human and mice tissues and may gain an immortalized phenotype. In contrast, primary cells and not immortalized cells can be cultured *in vitro* only to a certain degree [1]. Immortal cells either come from cancerous specimen or are engineered by using biotechnological techniques that e.g. prevent the DNA-telomeres of the cells from shortening [2].

Different cell lines that arise from one specific cell type frequently show similar biological properties. Thus, cell lines are standardized resources for *in vitro* research. Because standardization and reliability are the basis for biological and medical science, cell lines are distributed by cell banks. The most well-known cell bank probably is the American Type Culture Collection (ATCC).

In contrast to the standardization of cell lines, metadata describing these cell lines seem to be far more heterogeneous. Some effort has been made to extract the names of cell lines from the catalogs of cell banks and integrate them into biomedical ontologies [3], [4]. However, it is also important to know the cell lines' culturing conditions like growth medium, supplements, incubation temperature, and the corresponding protocols. After establishing new cell models in a laboratory, these factors are frequently adjusted according to the scientists' specific needs. However, it is important to record these adaptations in a controlled way, to ensure reproducibility of the respective experiments. While such information in general is available from the cell bank databases, it has only sporadically been included when cell line-specific ontologies were built.

In the past, many cell lines suffered from cross contamination, thus the experimental data were not suitable for further interpretation. As a consequence, within the last decade the authentication of cell lines came into the focus of researchers as well as major journals [5]. For example, a profound description of cell lines should include information for their identification, like short tandem repeat (STR) profiles [6].

For a transregional research network on liver cancer we identified the need to describe the assays and materials used by the 22 projects in a formalized way. Such a formal description enables the semantic integration of the data throughout the network. While there are many data types that need to be covered in a research network, for this study we focus on data related to cell culture experiments.

**Table 1:** List of cell banks compared.

Cell bank name	Internet address
American Type Culture Collection (ATCC)	http://www.atcc.org
European Collection of Cell Cultures (ECACC)	http://www.hpacultures.org.uk
Deutsche Sammlung von Mikroorganismen und Zellkulturen (DSMZ)	http://www.dsmz.de
Interlab Cell Line Collection (ICLB)	http://www.iclc.it
Riken	http://www.brc.riken.jp

The databases of five widely used cell banks were included into the comparison of data fields. The table shows the name of the Internet addresses were they can be reached. The Internet addresses were last accessed on March 1, 2012.

For data integration, the network uses a service oriented platform based on cancer Biomedical Informatics Grid (caBIG) [7], [8]. All research data are made available by means of data services. The data need to be annotated with a standardized set of metadata to facilitate the discovery of corresponding services and cross project data analysis. The resulting ontology will also be the basis for the computer based evaluation of the protocols created by different groups within the consortium. So far, it was not possible to link the assays and corresponding results across the network in an automated manner since the projects' experimental setup was not semantically annotated in a uniform way. By developing an ontology-based structure for protocols and their participants we will create an environment that will allow cross-project evaluation and direct comparison of experimental data.

**Table 2:** Ontology evaluation techniques (Obrst et al. [16]).

Technique	Description
Evaluate use of ontology in an application	Task-based evaluation is used to measure practical aspects of the ontology. Use-cases or scenarios can be used to characterize the target knowledge requirements.
Comparison of ontology against a source of domain data	The ontology is compared to other ontologies or databases of the corresponding knowledge domain.
Assessment by humans against a set of criteria	The ontology is assessed to see, if certain principles were considered during development. These criteria are often derived from common sense.
Natural language evaluation techniques	Ontologies are evaluated by measuring their impact on natural language processing tasks like knowledge extraction and question-answering.
Use reality as benchmark	Different versions of an ontology are measured by calculating a metric that describes the how good they correspond to the reality of the domain. This technique measures how good an ontology improves during the course of development cycles.
Ontology accreditation, certification, maturity model	Ontologies can be formally accredited and certified if standardized criteria and organizations are established for this task.

This table summarizes the evaluation techniques suggested by Obrst et al. [16] for use with ontologies in life sciences. The techniques one to three were applied when evaluating CCONT.

Cell lines are the main components for many experiments in biomedical research. Thus, in this article we focus on cell line-related aspects and create a basic ontology structure that can be used as a controlled vocabulary within a multi-project network. However, the use of the ontology is by no means limited to such research networks. In fact, it is a generic ontology that can be used for other cell line-related purposes as well. We not only want to reference the cell lines' identities, but also record their origin and propagation in a controlled way. When cell lines are used in cell culturing experiments they tend to develop aberrances in their properties. While these deviations are small they still might influence the comparability and reproducibility of experiments. Thus, users of the ontology will have to add individuals to represent these specific sub-strains of cell lines.

**Table 3:** Sample data fields in cell bank databases.

cell bank	‘medium’	‘organ’
ATCC	propagation*	source/organ
DSMZ	medium*	–
ECACC	culture medium*	tissue
ICLB	culture conditions*	description*
Riken	medium	tissue

The same information is attributed to different data fields in the cell banks we reviewed. This table shows how the two sample terms ‘medium’ and ‘organ’ are handled in the respective databases. The fields marked by * are free text fields. They are also used for other types of data.

In contrast to just storing the cell line data in a standardized database, we will be able to use the ontology to prepare the data about experiments for semantic integration with other kinds of research data and thus be able to deduce new research insights by processing the data with reasoning engines. Two examples for deducing knowledge from the ontology are the following:

**Table 4:** Sample data fields in cell bank databases.

data group	field name	MCCL	CLO	EFO
identification	cell line name	√	√	√
origin	ethnicity	×	×	√
	age	×	×	√
	sex	×	×	√
	species	√	×	°
	strain	×	×	°
	organ	√	×	√
	disease	√	×	√
cell line properties	growth mode	√	√	×
	morphology	√	√	√
	cellular products	×	×	√
	cytogenetics	×	×	×
	STR fingerprint	×	×	×
	biosafety level	×	×	×
	risk group	×	×	×
	viruses	×	×	×
	mycoplasma	×	×	×
propagation	medium	×	×	×
	supplements	°	×	°
	temperature	√	×	√
	atmosphere	×	×	√
	confluence rate	×	×	×
	seed density	×	×	×
	split ratio	×	×	×
	detachment aid	×	×	×

The table lists data elements derived from cell banks. The last three columns show, which of the cell line related ontologies have classes that cover the data elements: full coverage, 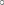 partial coverage, 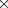 not covered.

- Identify cell culture conditions for a new assay based on the culture conditions of related cell lines.
- Identify cell line individuals that are suitable for a specific experiment based on former results of assays.

This leads us to the following questions, which we will answer in the course of this paper:

1. How are cell lines identified in cell bank associated databases?
2. Which data elements are essential for the description of cell lines and their culturing?
3. How should an ontology be designed that can act as a basis for the standardized description of cell lines?

**Figure 1:**
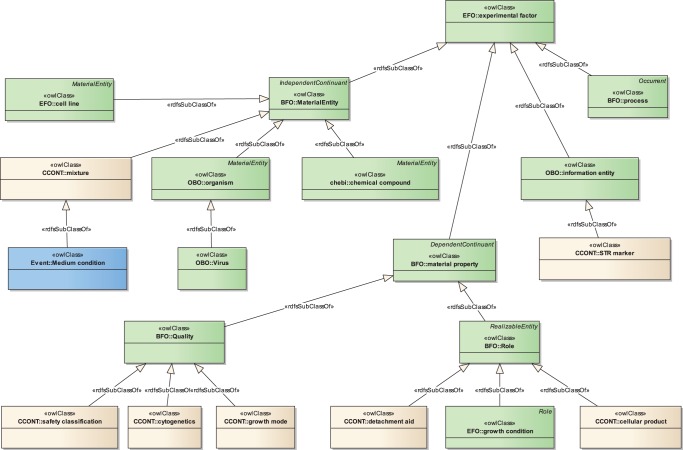
The structure of CCONT. This diagram shows some of CCONT's high-level classes to illustrate the structure of CCONT. The diagram is noted using the ontology profile of the Unified Modeling Language (UML, [40]). UML diagrams provide a standardized way to represent ontologies graphically [41]. Classes in green color are imported from the EFO ontology. The class ‘Medium condition’, colored in blue, represents the corresponding subtree imported from INOH's event ontology. Classes newly defined in CCONT are shown in beige. Note that the EFO ontology incorporates other ontologies like BFO or OBO. This is represented by a corresponding UML package name. To ensure the legibility of the diagram we only show subClassOf relations.

These questions will lead us to a domain specific ontology for cell lines. Its scope will be the representation of knowledge available on cell lines, related protocols, and growth conditions.

**Figure 2:**
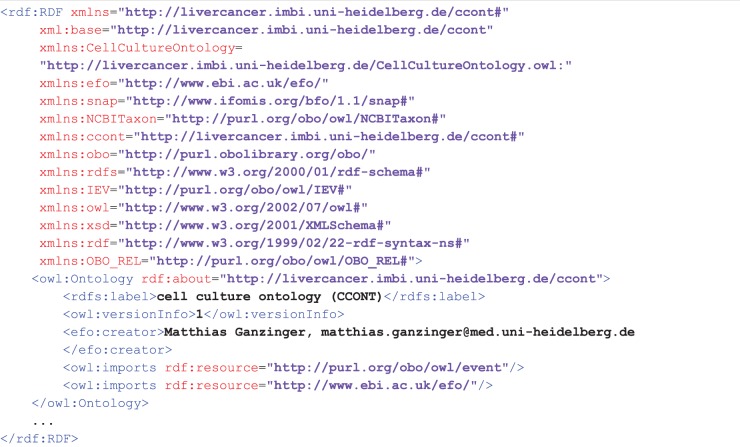
The head of CCONT's OWL file. Technically, for the definition of CCONT we started with a fresh OWL file. Following the considerations described in this article, we reused the two ontologies EFO and IEV's event ontology. This is reflected by corresponding XML namespace definitions and OWL import statements.

## Methods

For the development of our ontology we took the recommendations of Noy et al. [9] as a guideline. Thus, for a comprehensive description of cell lines we first need to analyze the necessary elements of metadata. We assume that the databases associated with major cell banks are suitable starting points for acquiring these metadata. Further, we conducted a literature research using PubMed and Google Scholar to determine the cell banks commonly used in biomedical research. As a result, we selected five large international cell banks as shown in Table 1. In addition, the Hyper Cell Line Data Base (HyperCLDB) exists as a collection of cell line information extracted from several databases [4]. Since the data structure available from HyperCLDB is only a subset of the original data, we decided not to include this database in our comparison.

**Figure 3:**
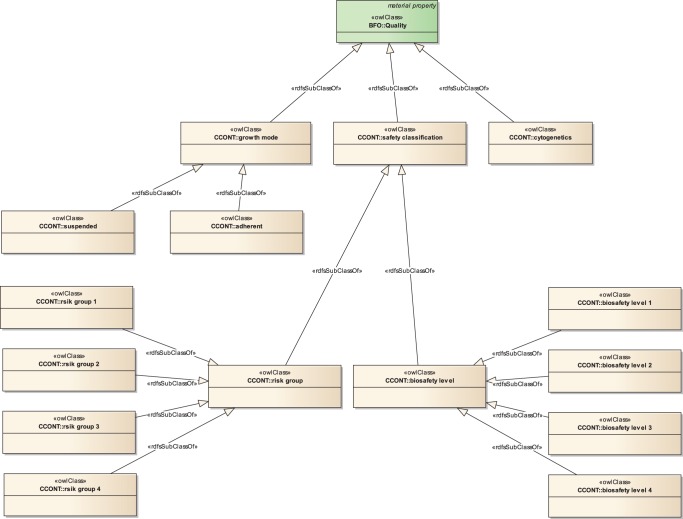
The ‘Quality’ subtree of CCONT. The ‘Quality’ class was originally defined as a child class to ‘material property’ in BFO. Since it is part of EFO it is also imported into CCONT. In CCONT we define several child-classes to ‘Quality’: ‘growth mode’, ‘safety classification’ and ‘cytogenetics’.

We extracted the data structures of cell bank catalogs available over the Internet. The data fields were compared and mapped to a consolidated name serving as a preliminary term. In some sources multiple concepts were put together in single free text fields. In these cases we split them into separate consolidated names. The consolidated names were grouped into different domains of cell line description. The groups were helpful to identify candidate ontologies, since those ontologies ideally have a distinct specialized scope.

**Table 5:** STR marker used in CCONT.

Amelogenin
CSF1PO
D13S317
D16S539
D18S51
D19S433
D21S11
D2S1338
D3S1358
D5S818
D7S820
D8S1179
F13A01
F13B
FESFPS
FGA
THO1
TPOX
vWA

The STR loci shown here are derived from the forensic STR system Combined DNA Index System (CODIS) [31]. Since the system is established to identify human cells with a high probability, it is also used by various cell banks for identifying human cell lines.

### Ontology development

Considering publications on ontology development methods [9], [10], we defined a four-step development process: define domain concepts; review of existing ontologies; construct conceptual model; provide formal representation.

**Figure 4:**
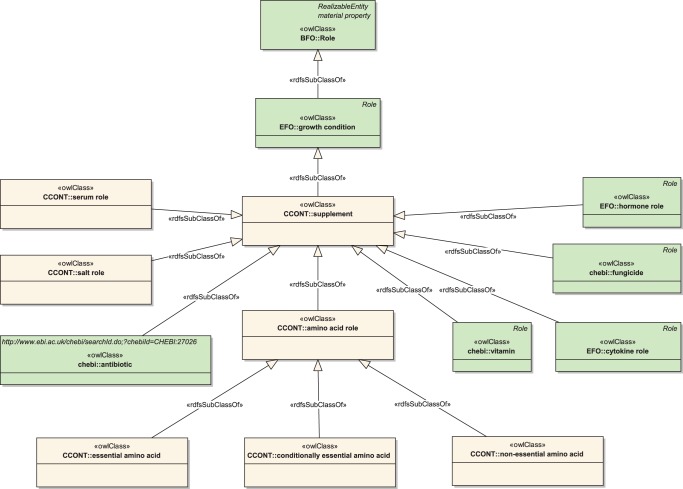
The ‘supplement’ subtree of CCONT. The class ‘supplement’ is defined in CCONT to identify substances that are necessary to support the growth of cell lines in culture. In other words, these substances are assigned specific roles in the process of cell culturing. As a consequence, we decided to place the ‘supplement’ class below EFO's ‘growth condition’ class in the ‘Role’ subtree.

#### Define domain concepts

The analysis of of the cell bank databases leads to the definition of the new ontology's conceptual domain and scope. Further, the names extracted from these databases are directly transformed into concepts of the conceptual domain. The intended ontology needs to represent all concepts of this domain, either by reusing an existing ontology or by newly defining the classes.

**Table 6:** Number of chemical compounds for each role in CCONT.

role	number of chemical compounds	
	total	imported from EFO
amino acid	20	0
antibiotic	19	1
cytokine	7	7
fungicide	3	1
hormone	13	5
salt	7	0
vitamin	1	1

Chemical compounds play different roles for the growth of cell lines. In column two we show how many compounds are necessary for describing a specific role. Column three shows the respective number of roles already present in EFO.

#### Review of existing ontologies

As a next step, ontologies covering the groups' domains were identified. This was done by research in Internet databases for biomedical ontologies. The best known might be BioPortal [11], which can be accessed under the URI http://bioportal.bioontology.org. As of March 2, 2012 it contains 302 ontologies with over 5.5 million terms in total. Another source we used is the Ontology Lookup Service (OLS) [12]. This database is accessible via the URI http://www.ebi.ac.uk/ontology-lookup (last accessed March 2, 2012). Our work was complemented by literature research via PubMed.

**Table 7:** Relationships imported from EFO.

Relationship	Description
bearer_of	A relation between an entity and a dependent continuant; the reciprocal relation of inheres insGOC:cjm] example of usage: red eye bearer of redness
contained_in	
has_quality	
has_role	A relation between a continuant C and a role R. The reciprocal relation of role of.
contains	
derived_into	
derives_from	Derivation as a relation between instances.
has_part	
has_participant	Has participant is a primitive instance-level relation between a process, a continuant, and a time at which the A continuant participates in some way in the process.
has_input	
inheres_in	
role_of	
is_executed_in	
is_realized_by	Relation between a realizable entity and a process. Reciprocal relation of realizes
located_in	
location_of	
part_of	
participates_in	Participates in is a primitive instance-level relation between a continuant and a process in which it participates. For example a scanner participates in a scanning process at some specific time.
is_input_of	
realizes	Relation between a process and a material fulfilling a role (i.e. realizing a role within the context of the process). For example a human realizing role of teacher within a lesson teching process.
regulates	
relationship	

In CCONT, we use the relationships inherited from EFO [21]. In this table we show the labels of the relationships and their description if available.

Candidate ontologies should have mappings for as many consolidated names identified in the previous step as possible. Since those ontologies were generally designed for their respective purpose and perhaps reuse but not specifically for cell lines, we did not expect to find perfect matches. Often, only parts of ontologies are useful and for some consolidated names it was likely not to find a predefined ontology at all.

**Table 8:** Cell lines used in the liver cancer research network.

type of cell line	cell line name
human cell line	Hep3B
	HLE
	HLF
	HuH7
	PLC/PRF-5
	HepaRG
	THLE2
	THLE3
	HepG2
mouse cell lines	Hep1-6
	Hep55.1C
	Hep56.1D
	BNL
primary cells	PHH
	PMH

To establish a common platform in the research network, these cell lines were chosen and procured centrally. These cell lines also serve as test items for CCONT.

**Table 9:** Representation of cell line Hep3B using CCONT.

Group	Identification	Classname	Value
identification	EFO_0002205	Hep3B	
origin	EFO_0003150	African American	
	EFO_0000246	age	8
	EFO_0001266	male	
	NCBITaxon_9606	Homo sapiens	
	EFO_0000887	liver	
	EFO_0000182	hepatocellular carcinoma	
properties	CCONT_0000081	adherent	
	CL_0000066	epithelial cell	
	CCONT_0000177	alpha-fetoprotein	
	CCONT_0000178	HBsAg	
	CCONT_0000102	cytogenetics	modal 60
	CCONT_0000100	Amelogenin	X
	CCONT_0000093	CSF1PO	8
	CCONT_0000090	D13S317	12,14
	CCONT_0000092	D16S539	10
	CCONT_0000089	D5S818	13
	CCONT_0000091	D7S820	8,1
	CCONT_0000086	THO1	6,7
	CCONT_0000096	TPOX	9
	CCONT_0000094	vWA	17
	CCONT_0000068	biosafety level 2	
	CCONT_0000179	EBV	negative
	CCONT_0000180	HBV	negative
	CCONT_0000181	HCV	negative
	CCONT_0000182	HIV	negative
	CCONT_0000183	HTLV-I-II	negative
	CCONT_0000184	SMRV	negative
	EFO_0000788	fungal component	negative
propagation	IEV_0000344	MEM(medium)	
	CCONT_0000048	fetal bovine serum	10
	EFO_0001702	temperature	37.0
	EFO_0000273	atmosphere	95%air
	EFO_0000273	atmosphere	5% co2
	CCONT_0000077	confluence rate	3
	CCONT_0000079	seed density	0.5E6
	CCONT_0000078	split ratio	1:4
	CCONT_0000076	detachment aid	trypsin

As an Example, we show how the liver cancer cell line Hep3B is represented by using CCONT. For each data element derived from a cell bank's database we evaluated if the data could be represented using CCONT's classes. In this specific example the data for the Hep3B cell line were extracted from the ATCC database.

The candidate ontologies were evaluated for their completeness and usability for cell line description. They were included into the project ontology. Ideally, the whole domain of cell lines could be covered just by combining previously defined ontologies. However, not all consolidated names could be mapped to an existing class.

#### Construct conceptual model

The ontologies imported for reuse as well as the newly defined classes are organized into a hierarchical structure. This conceptual model is the backbone of the new ontology since it ensures the reusability of the ontology e.g. by including an upper level ontology.

#### Provide formal representation

In a final step, the ontology is implemented in the Web Ontology Language (OWL) [13]. This representation is the basis for the evaluation of the ontology. The OWL-file is provided to the public for download over the Internet.

### Ontology evaluation

The new ontology needs to be evaluated for correctness and usefulness. Several approaches for evaluating ontologies can be found in literature [14], [15]. For the evaluation of this ontology we followed the techniques published by Obrst et al [16]. A summary of these techniques is provided in Table 2. As part of the evaluation we used nine human and four mouse cell lines as well as one primary human and one primary mouse cell type as test cases.

Further, we evaluated the consistency of the new ontology as suggested by Gómez-Pérez [17]. To ensure the axioms asserted in the ontology are free of contradictions, we classified the ontology using two different reasoners.

## Results

### Define domain concepts

The analysis of the cell banks (see Table 1) showed that they have different approaches for describing cell lines. While many information entities like the cell origin were present in all cell banks, these items were not always available in a structured way. ATCC for example has data elements called ‘Age’, ‘Gender’, and ‘Ethnicity’, but other cell banks like the European Collection of Cell Cultures (ECACC) have just one field called ‘cell line description’ that contains the same information as continuous text.

The analysis of the cell banks' databases also leads to the answer of our first question: As far as the identification of cell lines is concerned, there are similarities among the different databases. All cell banks provide a cell line name and an internal identification number. The cell line name might be available across different databases but inconsistencies may occur [3]. The identification number is only consistent within the specific database and not useful to correlate information across the databases.

In Table 3 the data elements ‘medium’ and ‘organ’ are shown as examples. For ‘medium’ only one cell bank provides a specific data field, while the others use free text fields containing multiple data entries. Most databases have distinct fields for the organs the cells are derived from, but the Deutsche Sammlung von Mikroorganismen und Zellkulturen (DSMZ) does not show the organ explicitly. For the complete analysis see the Supporting Information (Table S1). In total, we identified approximately 30 data elements, that were used to describe the cell lines. These data elements were related to one of five groups:

1. Identification: This group contains the cell line name used for identification.
2. Origin: These data elements describe the origin of the specimen from which the cell line was derived.
3. Cell line properties: This group contains data elements describing specific characteristics of a cell line.
4. Propagation: Metadata showing how to culture the cell lines are combined in this group.
5. Tests: Data elements about contamination tests are part of this group.

The data fields mapped to these groups are shown in the first two columns of Table 4. This table also summarizes the answer of our second question, as it shows the essential data elements for describing cell lines.

### Review of existing ontologies

In this section we show the results of our evaluation of existing ontologies relevant for the description of cell cultures. Since we did not find an ontology that covers all aspects of meta data discovered during the analysis of cell bank data sets, we discuss ontologies that cover the data element groups (cf. Table 4).

#### Cell line identification

As of March 1, 2012 BioPortal returns 17 ontologies that contain a class for the term ‘cell line’. In six of them the cell line class does not have subclasses. This means, these ontologies do not have classes for the well known cell lines already included, but have a placeholder where this information can be inserted. The overall structure of these ontologies might still be useful for the comprehensive description of cell lines. Since our goal was to reuse as much of existing ontologies as possible, we will focus on ontologies that contain classes for at least 100 cell lines.

There are two ontologies named ‘cell line ontology’. The first one, identified by the prefix MCCL, is available on BioPortal with id 1245 [18]. It includes the cell line names extracted from cell banks like ATCC. In addition, anatomical references to the Foundational Model of Anatomy (FMA) [19] are included as well as references to the Human Disease Ontology (DO) [20].

The other cell line ontology is abbreviated by the acronym CLO. The developers of CLO focused on the automated extraction and harmonization of cell line names from cell banks and databases [3]. The currently published version 2.0.27 on BioPortal has 8728 cell line names that are all subclasses of the class ‘permanent cell line’. CLO does not link to other concepts like anatomy.

Further, the Experimental Factors Ontology (EFO) [21], contains around 700 cell line names. Classes of cell lines have references to anatomic and disease information. Since this ontology is designed to describe biomedical experiments rather then cell lines alone, it has many classes that are necessary for a full set of cell line meta data.

#### Specimen origin

This group of meta data describes the origin of the sample from which the cell culture was established. It includes information about the species, like Homo sapiens or Mus musculus, but also more specific information like the donor's ethnicity or the strain of an animal model. In addition, the source organ of the specimen and the disease related to the cells need to be considered.

Of the ontologies already identified in the previous section, only EFO has classes for the description of the cell line's ethnic group. An alternative to EFO would be to use the terms from the Systematized Nomenclature of Medicine (SNOMED) Ethnic Groups http://bioportal.bioontology.org/ontologies/43057), which has 261 classes for the description of ethnic aspects.

While all cell line ontologies provide classes for sex, only EFO has one for age. EFO also has classes for cell line relevant species. The species support of MCCL is more comprehensive, since it also contains classes for animals less common in the laboratory. Strains of some model organisms are also part of EFO, especially for Mus musculus. As far as CLO and MCCL are concerned, only the cell lines derived from such model organisms are present, but not the species themselves.

All cell line ontologies mentioned in the previous section include anatomic classes for describing the source organs of the specimen that were imported from domain specific anatomy ontologies like FMA. Disease information is provided in all three ontologies by using the Disease Ontology.

#### Cell line properties

The cell line properties group of data elements describes the characteristics of the cells. It contains basic information necessary for handling and identifying the cell lines:

- *Growth mode* records whether the cells grow adherent to a surface or in suspension.
- *Morphology* describes the tissue type from which the cells are derived, e.g. epithelial or neuronal.
- *Cellular products* summarize the substances produced by the cells.
- *Cytogenetics* contains information about the cell lines' chromosomal properties.
- *STR fingerprint* is a set of numbers used to determine the authentity of human cell lines.
- *Bisosafety level and risk category* are used to classify hazardous agents.
- *Viruses and mycoplasma* describe for which contamination types the cells were tested.

While classes for morphology are present in all 3 cell line ontologies, EFO does not include a way to describe the growth mode. Cellular products are essentially chemical compounds and as such can be represented by EFO.

The term ‘cytogenetics’ appears in none of the cell line ontologies, but otherwise in 13 ontologies on BioPortal. All of these have a clinical background. Their cytogenetic classes mostly cover diseases originating from specific chromosomal aberrations. To describe the cytogenetic findings in a more general way, it would be helpful to follow the International System for Human Cytogenetic Nomenclature (ISCN) [22]. An ontology claiming to cover the description of chromosomal variation in a semantic network is the Clinical BioInformatics Ontology (CBO) [23].

The results for STR profiling are similar: The term is part of 3 ontologies on BioPortal, but none of these has subclasses for describing the actual profiles. To do this, classes for the markers currently used in human genetic identification should be present. Until a final standard for cell authentication is available [24], at least classes for markers used in forensic identification kits should be provided.

As of March 2, 2012 two ontologies contain terms to describe biosafety levels 1–4. BioAssay Ontology (BAO) [25] contains these classes as subclasses of ‘assay biosafety level’. Since we want to describe the properties of cell lines and not assays, the semantics used in BAO do not seem to match our needs. The second ontology dealing with biosafety is SNOMED Clinical Terms (SNOMED CT, http://bioportal.bioontology.org/ontologies/1353). In this case, the terms are independent of a specific application. Instead, the four terms are ordered under the term ‘Levels’ that is located below ‘Ranked categories’.

#### Cell propagation

This group is necessary to describe the conditions and processes for culturing the cells. It is important to know, which growth media, supplements and further environmental factors are necessary to provide optimized growth conditions for the cell culture.

All cell cultures need a specific growth medium that provides the cells with an optimized environment for cell propagation. These media are often prepared according to standardized formulas and can be ordered ready for use from biochemical companies. Unfortunately, none of the cell line ontologies has defined classes for common standardized media. However, we were able to find a comprehensive collection of cell culture media in the Integrating Network Objects with Hierarchies (INOH) Event ontology (IEV) [26].

Supplements are substances like salts or sera that are necessary for the cells to grow. These compounds usually have to be added to a cell culture in addition to the respective growth medium. CLO is not capable of representing such substances, MCCL has classes that cover some aspects of supplements. EFO has a subtree called ‘chemical compound’ that is suitable to cover many supplemental substances.

It is no surprise that the term ‘temperature’ plays a role in 104 ontologies, according to BioPortal. These ontologies include EFO, MCCL and the Ontology for Biomedical Investigations (OBI) [27]. Atmosphere is present in 15 ontologies, but EFO is the only cell line related ontology among these.

The remaining terms are related to the process of subcultivation. The expressions confluence rate, seed density, split ratio, and detachment aid were not reported by BioPortal to be present in any ontology.

### Construct conceptual model

In this section we will answer our third question regarding the design of a comprehensive ontology. To overcome the shortcomings of existing ontologies related to cell lines, we designed the Cell Culture Ontology (CCONT). We tried to satisfy the categories shown in Table 4 by reusing classes of existing ontologies as far as possible and only fill gaps remaining with more than 100 newly defined classes. Figure 1 shows the structure of CCONT in a condensed way.

We used the OWL-editing capabilities of Protégé [28] to develop the structure of CCONT. To allow interoperability with other ontologies, CCONT was chosen as a prefix to all class identifiers defined in this ontology. According to BioPortal, this prefix was not used by another ontology as of March 1, 2012. Each class has a unique identifier consisting of the prefix CCONT and a number incremented for each class, e.g. http://livercancer.imbi.uni-heidelberg.de/CCONT_0000001.

As a starting point, we decided to choose the ontology that covered most aspects of our intended domain. In our opinion, EFO fulfils this criteria, since it covers many aspects of different data element groups. Table 4 shows in the last column those fields of our evaluation that are supported by EFO. We imported EFO into CCONT by referencing its URI http://www.ebi.ac.uk/efo/. The latest version of EFO tested to work with CCONT is version 2.14, accessed July 27, 2011. All classes of EFO have unique identifiers containing the prefix EFO, e.g. http://www.ebi.ac.uk/efo/EFO_0000001.

In addition, we imported the classes describing culture media of the INOH event ontology. The classes of this ontology have unique identifiers using the prefix IEV, e.g. IEV_0000344. We imported IEVs subtree ‘medium conditions’ (class IEV_0000293) into CCONT. ‘Medium conditions’ was placed under the newly created class ‘mixture’, which is in turn a subclass of EFO's ‘material entity’ class.

Figure 2 illustrates the technical implementation of CCONT and how EFO and IEV were merged into CCONT. EFO provides the new root element ‘experimental factor’ (EFO 0000001). From the IEV ontology, only the ‘Medium conditions’ is used within CCONT. Thus, we used the following Resource Description Framework (RDF [29]) statements to place ‘Medium conditions’ under the class ‘mixture’ (CCONT 0000047):

<rdf:Descriptionrdf:about = "http://purl.org/obo/owl/IEV#IEV_0000293">

<rdfs:subClassOf

rdf:resource = "http://livercancer.imbi.uni-heidelberg.de/ccont#CCONT_0000047"/>

<rdfs:subClassOf>

<owl:Restriction>

<owl:onProperty rdf:resource = "http://purl.obolibrary.org/obo/OBI_0000316"/>

<owl:someValuesFrom rdf:resource = "http://www.ebi.ac.uk/efo/EFO_0000579"/>

</owl:Restriction>

</rdfs:subClassOf>

</rdf:Description>

For the data fields shown in Table 4 that were not covered by either EFO or IEV, we did not find suitable ontologies that we could reference in CCONT in a useful way. These classes were defined directly in CCONT. As far as the definition of the terms is concerned, we tried to make use of the Medical Subject Headings (MeSH, http://www.ncbi.nlm.nih.gov/mesh) or the Chemical Entities of Biological Interest (ChEBI) [30] database, where applicable.

The first element not covered by EFO or IEV is ‘growth mode’. We placed a corresponding class under the EFO class ‘Quality’ (EFO_0001436, see Figure 3) in the ‘material property’ tree. This field was originally defined in BFO. ‘Growth mode’ has the two subclasses ‘adherent’ and ‘suspended’.

The class ‘cellular product’ was placed beneath EFO's class ‘role’ (EFO_0001440), which is again a class inherited from BFO. The intention is to mark substances placed under ‘chemical compounds’ as a cellular product.

Due to the multitude of possible chromosomal aberrations, the comprehensive ontological representation of cytogenetics is challenging. The only ontology we found covering cytogenetics (CBO) cannot be partially integrated into CCONT because of licensing constraints. Since not all cell banks (ATCC being one of those) provide cytogenetic information in an ISNC conform way, we decided to define only a single class called ‘cytogenetics’ as a place holder in this version of CCONT. To facilitate reasoning on cytogenetic data, a future version of CCONT will have to implement an ISNC conform conceptualization of cytogenetic data. Textual information can be stored in a data property called ‘cytogeneticsProperty’.

For the description of STR fingerprints we created the class ‘STR marker’ below the class ‘information entity’ (EFO_0001435, cf. Figure 1). As subclasses we defined the loci shown in Table 5. These markers were chosen since they are part of forensic STR systems like the Combined DNA Index System (CODIS) [31]. They are also used in the Cell Line Integrated Molecular Authentication (CLIMA) database [4] and the ATCC cell bank.

Biosafety level and risk group are two concepts to describe the level of hazard emanating from the cell line in question. In CCONT, we defined a class called ‘safety classification’ under the class ‘quality’ (EFO_0001436) in the ‘material property’ tree as shown in Figure 3. It contains two subclasses ‘biosafety level’ and ‘risk group’. Each of these classes has four subclasses: We included ‘biosafety level 1′ to ‘biosafety level 4′ from SNOMED CT by referencing the corresponding SNOMED identifier. Further, we defined ‘risk group 1′ to ‘risk group 4′, according to the guidelines of the National Institutes of Health [32].

To represent tests for viruses in cell cultures we added 6 common virus types like HBV, HCV, or HIV as subclasses to the class ‘Virus’ (NCBITaxon_10239, see also Figure 1) that was already present in EFO. As far as mycoplasma is concerned, we decided to keep this as a free-text field, since a taxonomy describing fungal contamination has to be defined first.

In addition to the media conditions imported from IEV, classes for sera were defined as subclasses of ‘mixture’. The class ‘growth mode’ was placed as a subclass to ‘quality’, which was inherited from BFO by EFO. Most classes that had to be defined were for the description of supplements necessary for cell culture. As a container, we defined the class ‘supplement’ under ‘growth condition’ (EFO_0000523) in EFO's ‘role’ subtree. The structure of `supplement' and its children is illustrated in Figure 4. Note that some classes already present in EFO were linked to ‘supplement’ to indicate their role in cell culturing. The subclass structure of ‘supplement’ is as follows:

- ‘amino acid role’, with subclasses ‘essential’, ‘conditionally essential’, and ‘non-essential amino acid’.
- ‘antibiotic’ (from EFO_0001485).
- ‘cytokine role’ (from EFO_0003787).
- ‘fungicide’ (from EFO_0001823).
- ‘hormone role’ (from EFO_0001824).
- ‘salt role’.
- ‘serum role’.
- ‘vitamin’ (from EFO_0001831).

The supplement substances themselves are, with the exception of sera, located beneath the class ‘chemical compound’. They are connected to the specific role via the ‘has_role’ relation. Table 6 shows the number chemical compounds that were added to CCONT or imported from EFO with respect to their specific role. Sera are located under the class ‘mixture’ described earlier in this section. They also reference ‘serum_role’ by using the ‘has_role’ relation.

Confluence rate, seed density, and split ratio are measures that are important to provide the cells with ideal condition regarding the cell concentration in culture. We placed classes with corresponding names beneath EFO's class ‘measurement’ (EFO_0001444) in the ‘information entity’ tree of CCONT.

Apart from class definitions, relationships between classes are required. For CCONT, the relationships defined in EFO were used to represent the relationships among CCONT's concepts as well. EFO's relationships are shown in Table 7. Especially, the relationships ‘has role’, ‘has quality’, ‘is executed in’ and ‘participates in’ are important to cover the domain of CCONT. In addition, we defined the relationship has growth condition (CCONT 0000185) to describe the relation between cell cultures and growth conditions.

### Provide formal representation

CCONT is published as an OWL-file on BioPortal with the id 3108. It can be accessed under the URI http://bioportal.bioontology.org/ontologies/3108. BioPortal can be used to download and browse the ontology. Future releases of CCONT will be published on BioPortal as well.

### Evaluation of CCONT

Following the definition of Gómez-Pérez [17] we started with the verification of CCONT. Verification “refers to the technical activity that guarantees the correctness of an ontology” [17]. Thus, the consistency of CCONT was checked using two different reasoners available as plugins for Protégé. First, we used HermiT version 1.3.6 [33]. During the classification, no inconsistencies were reported by the HermiT reasoner. In Addition, we used the FaCT++ reasoner in version 1.5.3 [34]. Again, the reasoner fulfilled the classification process without any problems.

Gómez-Pérez defines validation as a process guarantees “that the ontologies [...] correspond to the systems that they are supposed to represent” [17]. To achieve this aim, we applied the techniques shown in Table 2 where possible:

#### Evaluate use of ontology in an application

To ensure the accuracy of CCONT we applied the ontology during the process of annotating the cell lines used in our research network (sample size n = 15). The names of these cell lines are shown in Table 8. As an example, we show the result of our annotation for the cell line Hep3B in Table 9. In this table, the classes and attribute values are documented for a specific individual, in this case the standard cell culture of Hep3B as shipped by ATCC. The representations of the remaining cell lines can be found in the Supporting Information (Table S2). They are documented analogous to Table 9. CCONT turned out to be sufficiently accurate to represent the intended domain of cell lines.

#### Comparison of ontology against a source of domain data

Due to the method we applied during development, CCONT is in accordance with previously developed ontologies covering the domain of cell lines. Further, the ontology covers knowledge of domain specific databases since it was derived from cell banks' databases listed in Table 1. Thus, we draw the conclusion that the ontology covers the intended domain in an adequate way.

#### Assessment by humans against a set of criteria

The authors assessed CCONT against the criteria derived during the development process (cf. section “cell bank data sets”).

#### Natural language evaluation techniques

CCONT was not validated using natural language processing (NLP) technology. This technique will be an interesting approach in the future e.g. when conventional protocols of assays are evaluated automatically.

#### Use reality as benchmark

Since CCONT is a newly developed ontology, we cannot compare it to previous versions. However, we consider this technique a useful approach when validating the next development cycle of CCONT.

#### Ontology accreditation, certification, maturity model

For now, we did not submit CCONT to any formal validation process. This might change in the future, e.g. when data are submitted to public metadata repositories that might require the certification of corresponding ontologies.

## Discussion

For the development of CCONT we analyzed, how cell lines are identified and what data fields are necessary to describe cell lines comprehensively in their context (Table 4). We also describe our suggestion for the design of an ontology for cell cultures.

Our approach is to reuse existing ontologies as far as possible, as also suggested by [9]. However, since there are many biomedical ontologies with overlapping domains we have to choose one that fits best to our needs. By choosing an ontology we also choose its structure, which may hamper the integration of other ontologies. It would be beneficial, if all biomedical ontologies would comply to the same upper level ontologies that could act as umbrella for ontology integration [35].

The Open Biomedical Ontologies (OBO) consortium [36] aims to coordinate the future integration of existing ontologies. EFO is not part of the OBO foundry or candidate ontologies, but it is based on a compatible upper level ontology, the Basic Formal Ontology (BFO) [37], [38]. This step should facilitate the interoperability with OBO ontologies. CLO includes OBI, which is based of BFO as well. MCCL on the other hand, does not include a reference to an upper level ontology.

An interesting adoption of CLO can be found in BAO. This ontology focuses on high-throughput screening and already includes classes to describe process aspects in cell culturing. However, the protocols, on which the assays are based, are not part of BAO but link to an external database.

At the International conference on Biomedical Ontology (ICBO) 2011 in Buffalo, NY, USA the maintainers of CLO and several other ontologies like BAO announced to work together to develop a comprehensive cell line ontology. This is a promising approach. However, as of March 1, 2012 the new version of CLO was not yet available to the public via BioPortal.

The process of developing and maintaining the ontology cannot be expected to be finished in the current state. In fact, CCONT will have to mature in future iterations of the process to be able to fulfill all the requirements for representing cell cultures. First steps will be the formalized representation of mycoplasma contamination tests, cytogenetics, and immunology. The formal description of the protocols used in cell culture assays will also be an important step in the development of CCONT. The Experiment ACTions (EXACT) ontology [39] was developed for the formal description of biomedical protocols for laboratory robots. While we do not focus on high-throughput technologies, EXACT might still be a suitable starting point for extending CCONT with protocol capabilities.

## Supporting Information

Table S1 **Comparison of data fields in cell bank databases.**
(PDF)

Table S2 **Representation of selected cell lines with CCONT.**
(PDF)
