# Supplementary material for: On the Ontology Based Representation of Cell Lines
Source: PLoS One. 2012 Nov 7;7(11):e48584. doi: 10.1371/journal.pone.0048584 (PMC3492450; doi:10.1371/journal.pone.0048584)
Supplement: Table S1 — Comparison of data fields in cell bank databases. (PDF) [file pone.0048584.s001.pdf]

## Comparison of data fields in cell bank databases

| Group | consolidated (CCONT) | description | ATCC | DSMZ | ECACC | ICLC | Riken |
|---|---|---|---|---|---|---|---|
| ID | cell line (name) | Name of cell line | Designations | Cell line: | Cell Line Name: | Cell line name | (table head) |
| Origin | Origin | origin of tissue cell line was establieshed from (e.g. 8-year-old black boy in 1976) | | Origin (freetext, used for multiple data entities) | see cell line description | | |
| | Species | Species of origin | Organism: | Species | Species: | see description | Animal |
| | Age | Age of origin | Age | see Origin | see cell line description | see description | Age |
| | Sex | Gender of origin | Gender | see Origin | see cell line description | see description | Sex |
| | Ethnicity | ethnicity (for human cell lines) | Ethnicity | see Origin | see cell line description | see description | Subspecies |
| | Strain | strain (e.g. mouse cell line) | Source / Strain | see Origin | see cell line description | | |
| | organ | | Source / Organ | | Tissue: | see description | tissue |
| | disease | | Source / Disease | Cell type: | Keywords | see description | Comment |
| cell line properties | Growth mode | cell growth mode (adherent, suspension) | Growth Properties: | | Growth Mode: | Morphology and growth | Anchoraged |
| | Morphology | Cell morphology (e.g. epithelieal) | Morphology: | Morphology | Morphology: | Morphology and growth | Morphology |
| | cellular products | substances produced by cells | Cellular Products: | see Origin | Products: | Properties | |
| | Cytogenetics | | Cytogenetic Analysis: | Cytogenetics | Karyotype: | | Chr distrb |
| | DNA fingerprint | | DNA Profile (STR): | Fingerprint | | Validation assays | |
| | bisosafetiy level | | Biosafety Level | Biosafety level | Cell Line Description | | |
| | Viruses | | | Viruses | | | Virus |
| | Mycoplasma | | | Mycoplasma | | Sterility | Mycoplasma |
| | | verbal description | | see Origin | Cell Line Description (freetext, used for multiple data entities) | Brief description (freetext, used for multiple data entities) | |
| | | verbal description | | | | Description (freetext, used for multiple data entities) | |
| Propagation | Medium | Medium for cell propagation | Propagation (freetext, used for multiple data entities) | Medium (freetext, used for multiple data entities) | Culture medium (freetext, used for multiple data entities) | Culture conditions (freetext, used for multiple data entities) | Medium |
| | Supplements | Supplements necessary for propagation | see propagation | see medium | see culture medium | see culture conditions | Antibiotics |
| | Temperature | Incubation temperature | Propagation / temperature | Incubation (freetext, used for multiple data entities) | see subculture routine | see culture conditions | Growth temp |
| | Atmosphere | Incubation atmosphere (air, %$CO_2$) | Propagation / atmosphere | see incubation | see subculture routine | see culture conditions | $CO_2$ concentration |
| | Subcultivation | Protocol for sub cultivation | Subculturing: | Subculture | Subculture Routine: | | Passage method |

| Color legend: |
|---|
| Field present in db |
| Field used for multiple purposes |
| Field not present |
