# Supplementary material for: On the Ontology Based Representation of Cell Lines
Source: PLoS One. 2012 Nov 7;7(11):e48584. doi: 10.1371/journal.pone.0048584 (PMC3492450; doi:10.1371/journal.pone.0048584)
Supplement: Table S2 — Representation of selected cell lines with CCONT. (PDF) [file pone.0048584.s002.pdf]

Group	Identification	Classname	Value
identification	EFO_0002205	Hep3B	
origin	EFO_0003150	African American	
	EFO_0000246	age	8
	EFO_0001266	male	
	NCBITaxon_9606	Homo sapiens	
	EFO_0000887	liver	
	EFO_0000182	hepatocellular carcinoma	
properties	CCONT_0000081	adherent	
	CL_0000066	epithelial cell	
	CCONT_0000177	alpha-fetoprotein	
	CCONT_0000178	HBsAg	
	CCONT_0000102	cytogenetics	modal number = 60
	CCONT_0000100	Amelogenin	X
	CCONT_0000093	CSF1PO	8
	CCONT_0000090	D13S317	12,14
	CCONT_0000092	D16S539	10
	CCONT_0000089	D5S818	13
	CCONT_0000091	D7S820	8,1
	CCONT_0000086	THO1	6,7
	CCONT_0000096	TPOX	9
	CCONT_0000094	vWA	17
	CCONT_0000068	biosafety level 2	
	CCONT_0000179	EBV	negative
	CCONT_0000180	HBV	negative
	CCONT_0000181	HCV	negative
	CCONT_0000182	HIV	negative
	CCONT_0000183	HTLV-I-II	negative
	CCONT_0000184	SMRV	negative
	EFO_0000788	fungal component	negative
propagation	IEV_0000344	MEM(medium)	
	CCONT_0000048	fetal bovine serum	10
	EFO_0001702	temperature	37.0
	EFO_0000273	atmosphere	95%air
	EFO_0000273	atmosphere	5% co2
	CCONT_0000077	confluence rate	3
	CCONT_0000079	seed density	0.5E6
	CCONT_0000078	split ratio	1:4
	CCONT_0000076	detachment aid	trypsin

Group	Identification	Classname	Value
identification	CCONT_0000187	HUH-7	
origin	EFO_0003164	Japanese	
	EFO_0000246	age	57
	EFO_0001266	male	
	NCBITaxon_9606	Homo sapiens	
	EFO_0000887	liver	
	EFO_0000182	hepatocellular carcinoma	
properties	CCONT_0000081	adherent	
	CL_0000066	epithelial cell	
	CCONT_0000100	Amelogenin	X
	CCONT_0000093	CSF1PO	11
	CCONT_0000090	D13S317	10
	CCONT_0000092	D16S539	10
	CCONT_0000089	D5S818	12
	CCONT_0000091	D7S820	11
	CCONT_0000086	THO1	7
	CCONT_0000096	TPOX	8,11
	CCONT_0000094	vWA	16,18
	CCONT_0000179	EBV	negative
	CCONT_0000180	HBV	negative
	EFO_0000788	fungal component	negative
propagation	IEV_0000304	DMEM(medium)	
	CCONT_0000048	fetal bovine serum	10%
	EFO_0001702	temperature	37.0
	EFO_0000273	atmosphere	95%air
	EFO_0000273	atmosphere	5% co2
	CCONT_0000079	seed density	0.5E5
	CCONT_0000078	split ratio	1:4
	CCONT_0000076	detachment aid	trypsin

Group	Identification	Classname	Value
identification	CCONT_0000188	PLC-PRF-5	
origin	NCBITaxon_9606	Homo sapiens	
	EFO_0000887	liver	
	EFO_0000762	hepatocellular hepatoma	
properties	CCONT_0000081	adherent	
	CL_0000066	epithelial cell	
	CCONT_0000178	HBsAg	
	CCONT_0000100	Amelogenin	X
	CCONT_0000093	CSF1PO	10
	CCONT_0000090	D13S317	11,12
	CCONT_0000092	D16S539	13
	CCONT_0000089	D5S818	12
	CCONT_0000091	D7S820	9,11
	CCONT_0000086	THO1	7,8
	CCONT_0000096	TPOX	8
	CCONT_0000094	vWA	15,16
	CCONT_0000068	biosafety level 2	
propagation	IEV_0000304	DMEM(medium)	
	CCONT_0000048	fetal bovine serum	10
	CHEBI:28300	glutamine	2mM
	EFO_0001702	temperature	37.0
	EFO_0000273	atmosphere	95%air
	EFO_0000273	atmosphere	5% co2
	CCONT_0000077	confluence rate	3
	CCONT_0000079	seed density	0.2E5
	CCONT_0000078	split ratio	1:4
	CCONT_0000076	detachment aid	trypsin

Group	Identification	Classname	Value
identification	EFO_0001186	HepaRG	
origin	EFO_0001272	adult	
	EFO_0001265	female	
	NCBITaxon_9606	Homo sapiens	
	EFO_0000887	liver	
	EFO_0000182	hepatocellular carcinoma	
properties	CCONT_0000081	adherent	
	CL_0000066	epithelial cell	
	CCONT_0000180	HBV	negative
	CCONT_0000181	HCV	negative
	CCONT_0000182	HIV	negative
	EFO_0000788	fungal component	negative
propagation	EFO_0001702	temperature	37.0
	EFO_0000273	atmosphere	95%air
	EFO_0000273	atmosphere	5% co2

Group	Identification	Classname	Value
identification	CCONT_0000193	THLE-2	
origin	EFO_0001272	adult	
	NCBITaxon_9606	Homo sapiens	
	EFO_0000887	liver	
	EFO_0000182	hepatocellular carcinoma	
properties	CCONT_0000081	adherent	
	CL_0000066	epithelial cell	
	CCONT_0000102	cytogenetics	near diploid
	CCONT_0000068	biosafety level 2	
	EFO_0000788	fungal component	negative
propagation	IEV_0000356	BEGM (medium)	
	CCONT_0000048	fetal bovine serum	10
	EFO_0001702	temperature	37.0
	EFO_0000273	atmosphere	95%air
	EFO_0000273	atmosphere	5% co2
	CCONT_0000078	split ratio	1:5
	CCONT_0000076	detachment aid	trypsin

Group	Identification	Classname	Value
identification	CCONT_0000192	THLE-3	
origin	EFO_0001272	adult	
	NCBITaxon_9606	Homo sapiens	
	EFO_0000887	liver	
	EFO_0000182	hepatocellular carcinoma	
properties	CCONT_0000081	adherent	
	CL_0000066	epithelial cell	
	CCONT_0000102	cytogenetics	near diploid
	CCONT_0000100	Amelogenin	X
	CCONT_0000093	CSF1PO	11,12
	CCONT_0000090	D13S317	13
	CCONT_0000092	D16S539	11,12
	CCONT_0000089	D5S818	13
	CCONT_0000091	D7S820	8,1
	CCONT_0000086	THO1	8,9,3
	CCONT_0000096	TPOX	6,9
	CCONT_0000094	vWA	17,18
	CCONT_0000068	biosafety level 2	
	EFO_0000788	fungal component	negative
propagation	IEV_0000356	BEGM (medium)	
	CCONT_0000048	fetal bovine serum	10
	EFO_0001702	temperature	37.0
	EFO_0000273	atmosphere	95%air
	EFO_0000273	atmosphere	5% co2
	CCONT_0000078	split ratio	1:5
	CCONT_0000076	detachment aid	trypsin

Group	Identification	Classname	Value
identification	EFO_0001187	HepG2	
origin	EFO_0003156	Caucasian	
	EFO_0000246	age	15
	EFO_0001266	male	
	NCBITaxon_9606	Homo sapiens	
	EFO_0000887	liver	
	EFO_0000182	hepatocellular carcinoma	
properties	CCONT_0000081	adherent	
	CL_0000066	epithelial cell	
	CCONT_0000177	alpha-fetoprotein	
	CCONT_0000102	cytogenetics	modal number = 55
	CCONT_0000100	Amelogenin	X,Y
	CCONT_0000093	CSF1PO	10,11
	CCONT_0000090	D13S317	9,13
	CCONT_0000092	D16S539	12,13
	CCONT_0000089	D5S818	11,12
	CCONT_0000091	D7S820	10
	CCONT_0000191	F13A01	5,7
	CCONT_0000190	F13B	6,1
	CCONT_0000189	FESFPS	11
	CCONT_0000086	THO1	9
	CCONT_0000096	TPOX	8,9
	CCONT_0000094	vWA	17
	CCONT_0000068	biosafety level 2	
	CCONT_0000179	EBV	negative
	CCONT_0000180	HBV	negative
	CCONT_0000181	HCV	negative
	CCONT_0000182	HIV	negative
	CCONT_0000183	HTLV-I-II	negative
	CCONT_0000184	SMRV	negative
	EFO_0000788	fungal component	negative
propagation	IEV_0000344	MEM(medium)	
	CCONT_0000048	fetal bovine serum	10
	EFO_0001702	temperature	37.0
	EFO_0000273	atmosphere	95%air
	EFO_0000273	atmosphere	5% co2
	CCONT_0000078	split ratio	1:4
	CCONT_0000076	detachment aid	trypsin

Group	Identification	Classname	Value
identification	CCONT_0000194	Hepa 1-6	
origin	EFO_0001754	strain or line design	C57L
	NCBITaxon_10090	Mus musculus	
	EFO_0000887	liver	
	EFO_0000762	hepatocellular hepatoma	
properties	CCONT_0000081	adherent	
	CL_0000066	epithelial cell	
	CCONT_0000067	biosafety level 1	
propagation	IEV_0000304	DMEM(medium)	
	CCONT_0000048	fetal bovine serum	10
	EFO_0001702	temperature	37.0
	EFO_0000273	atmosphere	95%air
	EFO_0000273	atmosphere	5% co2
	CCONT_0000078	split ratio	1:4
	CCONT_0000076	detachment aid	trypsin

Group	Identification	Classname	Value
identification	CCONT_0000195	BNL	
origin	EFO_0001754	strain or line design	BALB/c
	NCBITaxon_10090	Mus musculus	
	EFO_0000887	liver	
	EFO_0000761	normal	
properties	CCONT_0000081	adherent	
	CL_0000066	epithelial cell	
	CCONT_0000067	biosafety level 1	
propagation	IEV_0000304	DMEM(medium)	
	CCONT_0000048	fetal bovine serum	10
	EFO_0001702	temperature	37.0
	EFO_0000273	atmosphere	95%air
	EFO_0000273	atmosphere	5% co2
	CCONT_0000078	split ratio	1:4
	CCONT_0000076	detachment aid	trypsin
